# Annotations

**Published:** 1888-03-17

**Authors:** 


### ANNOTATIONS.

**The World and its Pedagogues.**

THE human soul is a much-harassed toiler in intellectual and moral fields. Busy and fussy pedagogues, ignorant or unmindful of the daily and strenuous tasks it has to perform in those regions which are objective and properly external to itself, are always making attempts to catch it, and pound and pummel and lick it into a shape which they are pleased to denominate "cultured." The whole object in life for living souls, according to those spectacled schoolmasters, is not *to do something*, but *to be cultured*. The *Spectator*, which is not altogether without insight into the religions both of the Old and New Testaments, permits this fundamental error to hold an honoured place in its pages, as if no other view had ever presented itself either to the editorial or to the scriptorial mind. Sir James Paget is made for the moment the champion of what is termed "scientific culture," and the *Spectator's* contributor constitutes himself the opposing counsel on behalf of "literary culture." Is, then, the whole end and object of man's existence in the world his "culture," or is not culture properly a reflex result of the performance of duty —work with a purpose and for recognised results? It seems to us that the *Spectator's* position is Greek, not Hebrew or Christian, and is in itself fundamentally and radically lower and less final than the diviner standpoints of the Old and New Testaments. It is, to the present writer at least, a very striking circumstance that the book of Genesis records of the Almighty that almost the earliest commandment he gave to man contained this prime idea of work upon external objects as being the highest duty of man. The command to man was, "Be fruitful and multiply, and *replenish the earth and subdue it.*" The Old Testament throughout proceeds on the same main lines. Culture, form, are not put in the foreground at all. The New Testament does not depart in the least from this clearly-defined position. There is still the same view of life. The perfectly intelligent and efficient soul is to pursue its work in objective regions. Does this, then, mean that culture is naught? Certainly not! It is a result, but not the primary and chiefly-intended result; and the culture which results from the carrying out of the Hebrew and the Christian ideals is in every way higher than that which grows from culture sought for its own sake. We can understand Mr. Matthew Arnold's championship of Greek inspiration, but not the *Spectator's*.

**An Experimental Test.**

THE large world, which is really of more actual value than the philosophers, in spite of the hard names they have so freely bestowed upon it, has certain rough and ready methods of finding out the worth of a thing. Sir James Paget, Huxley, and many others praise highly what is called scientific culture. A multitude which no man can number, who have had the benefit of public schools and universities, and many of whom, in addition to a little classical and less mathematical knowledge, have been given to understand that the Greeks had ideas, and that their ideas have come to us with certain glosses added at different intellectual periods by more or less subtle schoolmen and philosophers—this vast, and for the most part undistinguished, multitude, which does not possess even an elementary knowledge of science, is pleased to swear by literary culture. The world—not without authority—is apt to judge a tree by its fruits. Whether of the two "cultures," it asks, has been most useful, the scientific or the literary? We are not including with the literary the Hebrew and Christian. Comparing the purely literary with the purely scientific, we are bound to express the conviction that for the highest purposes of human life the value of science exceeds that of literature immeasurably. Leaving entirely out of account such hard and practical facts as "the steamship and the railway," and all the discoveries of chemists, physiologists, and astronomers, we make bold to affirm that science has beaten literature even on its own ground. The triumphs of literature should be, as they mostly have been, in the intellectual region; but that highest result of all culture, the full and complete emancipation of the human mind, was due to science and to science alone. We have but to mention Bacon, and a host of others who preceded and followed him, to be reminded at once that from the time of that most clear intellect there have always been men who, regardless of custom, authority, and danger, have stood up fearlessly to see what they could see with their own single eye, and to proclaim what they saw, and nothing else, with their own unconventional tongue. This result of the final and irretrievable destruction of the intellectual Bastille is what the world sees science to have brought about; and having regard to its own well-being and advancement towards that which is most human and best, it gives the palm, for the moment at least, to the champion of popular rights. Tested by this standard of fruit, science is golden, whilst mere literature is not higher in the scale than silvern, if indeed it be so high.

**Can a Man of Science Wonder?**

IN estimating the value of literary as compared with scientific culture, the influence of each upon personal character must be intelligently considered. As we have before stated, literary culture is not to be regarded as synonymous with moral and religious training. It is impossible in a brief note to deal broadly with the subject; but one particular point, which is made much of by the *Spectator*, affords an opportunity for contrasting the intellectual and moral fruits of the two. According to Sir James Paget, with whom the *Spectator* concurs, the faculty of *wonder* in the scientific man diminishes in proportion to his victorious progress in knowledge and power. If that be universally so, we are disposed to admit that science as a teacher is defective in at least one important element of culture. But is it so? Sir James Paget, who deservedly occupies a high place among thoughtful and scientific men, perhaps hardly does justice to science in this respect. It could be shown without much difficulty by many examples that, so far from this being universally or even largely the case, many scientific men as they have advanced step by step in knowledge—seeing deeper and deeper into the facts and processes and laws, into the resistless might and stored potentialities of the world of life and sense in which they find themselves —have become overwhelmed almost even to madness by the incomprehensible and the unsearchable which still lay beyond their ken. No one will contend that Goethe, who belonged to the two republics of science and letters, was destitute of wonder. Did the world of facts and of men, or the world of literature fill him most with awe? It was surely the world of facts and of men! May not Ruskin be truly called a man of science, even though he be not one in the narrow specialist's sense? Who is there among us so filled and penetrated with the mystery and miracle of the unknown and the unfathomable as he? Not indeed with what he calls "a mean wonder, as of a child who sees a juggler tossing golden balls," which he says "is base if you will. But do you think that the wonder is ignoble, or the sensation less, with which *every human soul* is called to watch the golden balls of heaven tossed through the night by the hand that made them?" The mere specialist, be his department what it will, may indeed, like the minnow in his pool, make his specialty his world; and when he has mastered the facts which his scalpel or his microscope or his calculating machine can reveal to him, he may possibly cease to be awed. But, if there be a man who, having reached such a stage, has no power to project his imagination further into the unknown, does he really deserve to be called a scientific man at all? It seems to us that to a mind of any breadth and capacity a very little of the knowledge which science reveals must make the world new and living as it could never have been before, whilst a deeper and broader vision cannot but fill him with a sense of even more than the Hebrew Psalmist's overwhelmed insignificance— "When I consider thy heavens, the work of thy fingers, the moon and the stars, which thou hast ordained—What is man?"

